# Political Uncertainty and the Timing of Mass Layoffs

**DOI:** 10.1177/10591478251331149

**Published:** 2025-04-02

**Authors:** Varouj Aivazian, Tzu-Ting Chiu, Miguel Minutti-Meza, Dushyantkumar Vyas

**Affiliations:** 1 Department of Economics and Rotman School of Management, 7938 University of Toronto, Toronto, ON, Canada; 2 Department of Accounting, Auditing and Law, NHH Norwegian School of Economics, Bergen, Norway; 3 Miami Herbert Business School, 5452 University of Miami, Coral Gables, FL, USA; 4 Department of Management – UTM and Rotman School of Management, 7938 University of Toronto, Toronto, ON, Canada

**Keywords:** Political Uncertainty, Layoffs, Labor Adjustment, Cost Restructuring, Real Options

## Abstract

This study examines the relation between political uncertainty arising from state-level election cycles and the timing of employee dismissal and plant closure notices filed by US firms under the Worker Adjustment and Retraining Notification (WARN) Act of 1988 (hereafter, WARN notices). We appeal to a real options framework to predict that firms delay layoff decisions and the issuance of WARN notices until the resolution of political uncertainty. Using establishment-level data on layoffs disclosed in WARN notices and state elections occurring between 1994 and 2022, we document that the likelihood of issuing WARN notices declines during the election quarter but increases in the subsequent quarter. Cross-sectional findings show that political uncertainty plays a significant role in the timing of WARN notices during election periods while other factors, including partisanship, economic conditions, union strength, and firm visibility, may also play a role. Further, firms that delay WARN notices do not experience a significant deterioration in their medium-term financial performance. Overall, our findings provide evidence that firms delay labor adjustment decisions and the announcements of such decisions in response to political uncertainty.

## 1. Introduction

Future government policies are unpredictable and shift over time with political turnover. This study examines the effect of political uncertainty on firms’ cost restructuring decisions. Specifically, we study the relation between increased political uncertainty during state-level election cycles and the timing of employee dismissal and plant closure notices filed by the US firms under the Worker Adjustment and Retraining Notification (WARN) Act of 1988. The WARN Act is a US labor law that protects employees, their families, and communities by requiring employers with 100 or more employees to provide a 60 calendar-day advance notice of plant closings and mass layoffs. The advance notice is intended to give workers time to adjust to the loss of employment, to look for and to obtain other employment and, if necessary, to enter skill training or retraining programs.

A growing literature in operations management, economics, finance, and accounting documents that political uncertainty affects economic outcomes and corporate decisions (see a review of this literature in Section 2). This literature is based on the premise that political uncertainty increases around election periods in the US and other jurisdictions. Although a precise definition of political uncertainty remains elusive, Pástor and Veronesi (2012) characterize it as uncertainty about the government's future actions. Political uncertainty is linked to a variety of economic and regulatory factors, such as consumer preferences, demand uncertainty, investment incentives, minimum wage laws, safety and environmental laws, and tax rates (Aaberge et al., 2017; Bernanke, 1983; Jens, 2017; Lee, 2018; Lee et al., 2020; Li and Zhang, 2024). To influence corporate investment and cost decisions, these factors must influence managers’ perceived cash flows and discount rate risks and ultimately their expected payoffs on corporate projects.

Our study focuses on firms’ decisions to announce a layoff of a large number of employees within the same facility in the presence of political uncertainty arising from the cycles of state-level elections. A similar setting has been previously used to show that political uncertainty delays various forms of investment (e.g., Blake and Moschieri, 2017; Çolak et al., 2017; Jens, 2017). Large layoffs can result in significant savings in fixed costs, improved productivity, and enhanced corporate focus. However, large layoffs are economically and strategically significant actions that are difficult to reverse, and their outcome is subject to increased uncertainty during election periods.^1^

We use a real options framework and an intertemporal flexibility decision model to support our prediction that firms deal with increased uncertainty by delaying downsizing decisions and the announcements of such decisions. The insights derived from the real options framework are implicit in the literature on political uncertainty; for instance, Bernanke (1983) predicts that the uncertainty of an election increases the value of postponing investments. In our model, we focus on demand uncertainty, which is a factor emphasized by Lee et al. (2020) as a driver of firms’ asymmetric cost responses to the changes in activity during periods of high political uncertainty surrounding national elections across countries. As noted by Giroud and Mueller (2017) and Lee et al. (2020), if companies expect a temporal change in demand, they are likely to retain workers until uncertainty is resolved to avoid dismissal, hiring, and training costs. Moreover, Boutchkova et al. (2012) argue that domestic politics influence labor legislation as politicians change labor laws in favor of or against employees, thereby affecting potential labor adjustment costs. Finally, existing research in management accounting suggests that managers are more likely to choose a flexible cost structure when they face uncertain demand (Holzhacker et al., 2015a, 2015b).

Anecdotal and archival evidence suggests that managers, politicians, and employees recognize increased levels of uncertainty during election periods. For example, in the 2024 US election cycle, issues about diversity, equity, and inclusion (DEI) and the loss of manufacturing jobs were frequently mentioned by candidates for local, state, and federal positions. In the United States, state legislatures oversee a broad range of important policies. During the 2020 election cycle, Florida's Amendment 2, which raised the minimum wage in the state, made national headlines.^2^ Notably, in contested state election cycles, a small group of informed or organized voters could determine the outcome when many other voters may be uninformed or have diverse preferences. Fouirnaies and Hall (2022) examine 780,000 bills and voting records for roughly 6000 US state legislators with term limits and document that state legislators appear to worry about their reelection prospects and consequently allocate more of their time toward visible dimensions of productivity when they run for reelection.

Our examination of election cycles for 47 states with WARN data available between 1994 and 2022 documents a pattern of short-term delays in layoff decisions and their announcements. In our univariate analysis, we find that the likelihood of WARN notices in our sample increases from 2.1% during the election quarter (2.2% in the quarter preceding the election) to 2.7% in the quarter following the election. Our multivariate regression analyses confirm that the likelihood of issuing WARN notices declines significantly during the election quarter but increases in the next quarter. We also find a similar pattern in the size of layoffs, measured by the number of affected employees as disclosed in WARN notices.

We perform cross-sectional analyses to examine key sources of variation in uncertainty and other factors that support or extend our main inferences. We find that the delay in WARN notices increases when (1) political risk and policy uncertainty are high, (2) the same party holds state or legislative control, (3) firms are politically connected, (4) local economic conditions are poor, (5) unions are weak in a state, and (6) firms are larger. Taken together, these cross-sectional findings indicate that political uncertainty drives our main results while other factors, including partisanship, economic conditions, union strength, and firm visibility, may also influence the timing of WARN notices during election periods.

We conduct additional analyses to (1) control for the effect of gubernatorial elections, (2) investigate whether the delay in WARN notices affects future firm performance, and (3) provide descriptive evidence on whether the patterns of layoffs with versus without WARN notices differ around elections. We find that (1) state-level election cycles involving governor appointments do not exhibit a significant incremental effect on WARN notice issuance, (2) there are no significant differences in financial performance, measured by return on assets (ROA) over the seven quarters following the election quarter, between delayers and nondelayers, and (3) some firms might lay off employees without WARN notices during the election quarter to avoid attention.

Overall, our study contributes to the literature on the consequences of political uncertainty (e.g., Bird et al., 2023; Julio and Yook, 2012; Jens, 2017; Lee et al., 2020; Leung and Sun, 2021; Mekhaimer et al., 2024). A pervasive finding in this literature is that firms delay investments until political uncertainty is partially or fully resolved. Our study extends the existing research by analyzing the impact of political uncertainty on the timing of layoff announcements. To the best of our knowledge, we are the first to examine the timing of WARN notices—an important feature of the US labor market—in the context of political uncertainty.

Our predictions and findings are related to those of Lee et al. (2020), who document increased asymmetry between changes in sales and total operating expenses (i.e., increased cost stickiness) during national election years across countries, suggesting that managers retain slack resources when political uncertainty is high. While both Lee et al. (2020) and our study focus on postponing cost reductions during election cycles, our study differs from theirs in three key aspects. First, by examining layoff notices at the state level, our findings link increased uncertainty caused by local elections to delays in substantial reductions in employment. A decrease in total operating expenses captures employment changes only indirectly, and recent evidence from Atanassov et al. (2024) shows that certain costs, such as research and development (R&D) expenses, may be accelerated when uncertainty is high. Additionally, WARN notices are filed in advance of actual layoffs and are therefore timelier and more relevant than financial reports. Cost reductions associated with layoff decisions may take several quarters to materialize, and accounting rules on the recognition of termination and restructuring costs make it difficult to pinpoint when a layoff decision was made. Furthermore, multinational firms may respond differently to heightened uncertainty from national elections, depending on the composition of their global workforce. Second, our research does not focus on asymmetric cost responses to sales declines, as some firms may lay off workers without immediate sales decreases—either after a sustained decrease in sales or prospectively when expected demand is low. Third, using US state election cycles, our findings align with the uncertainty and delayed investment setting of Jens (2017).

Our study also contributes to the operations management literature by providing evidence on how firms manage labor costs in business operations during periods of high political uncertainty. Our results complement the evidence presented by Leung and Sun (2021) on how firms spread their revenue streams among different customers in response to heightened uncertainty. Additionally, our study speaks to the broader literature on layoff and divestiture decisions (e.g., Blake and Moschieri, 2017; Falato and Liang, 2016). Our findings complement the study by Prabowo et al. (2018), which examines a sample of 22 European countries and finds that state-owned enterprises are reluctant to undertake downsizing decisions and exhibit greater labor cost stickiness compared to private firms. Finally, our study is related to research on strategic employment/layoff and disclosure decisions during election cycles (e.g., Bertrand et al., 2018; Piotroski et al., 2015), as it documents delays in firms’ layoffs and issuances of WARN notices in state election quarters.

## 2. Institutional Background and Relevant Literature

### 2.1. WARN Notices as a Means of Systematically Identifying Large Layoffs in the United States

We study employee dismissal and plant closure notices pursuant to the WARN Act of 1988. These notices are important to employees and their representatives, shareholders, creditors, politicians, and other interested parties in the local economy. The WARN Act requires employers to issue 60-day advance notices to affected employees, state-level chief elected officials, and relevant state-level labor departments (Acharya et al., 2013; Levine, 2007). The issuance of a WARN notice constitutes a material event and simultaneously prompts an 8-K filing by SEC registrants under Item 2.05 “Costs Associated with Exit or Disposal Activities” or Item 7.01 “Regulation FD Disclosure.”^3^

WARN notices constitute a primary way to identify substantial changes in a company's workforce at the state level for several reasons. First, failure to give layoff notice can result in penalties. If an employer does not give workers 60 days’ notice for a qualifying WARN event, they are liable to each employee for back pay and benefits for the period of violation, up to 60 days. In addition to the noncompliance penalty, the employer is subject to a civil penalty of at most $500 per day, but this fine may be waived if the employer settles liabilities with employees promptly. Moreover, recent proposed legislation, the Fair Warning Act of 2019, sought to amend the WARN Act to increase its scope and coverage (U.S. Congress, 2019).

Second, beyond federal legislation, several states have introduced additional penalties and enforcement mechanisms. For example, in 2008, New York enacted a state WARN Act that requires employers with 50 or more employees to give a 90-day notice for layoffs affecting 25 or more employees. A lack or delay of a WARN notice can result in litigation costs, as workers, their representatives, or local government units may file cases in federal and state courts.

Third, firms that issue WARN notices typically follow through with eventual employee dismissals. Issuing a WARN notice might reveal unfavorable news about a firm's financial health to shareholders and employees. A WARN notice may encourage some workers, especially the most productive ones with outside options, to seek new employment (Schwerdt, 2011). Descriptive data from July 1996 to December 2019 in Krolikowski and Lunsford (2024) indicates that the median number of days between a WARN notice date and the anticipated layoff date is usually no less than 60 days, aligning with the advance notice period required by the WARN Act. The advance notice distribution is skewed to the left: the 75th percentile rarely exceeds 90 days, while the 25th percentile often falls to about 30 days, and sometimes close to 0 days. Evidence in Krolikowski and Lunsford (2024) shows that WARN notices lead state-level initial unemployment insurance claims, changes in the unemployment rate, and changes in private employment. Broadly, they conclude that WARN data is a useful indicator of aggregate job loss.

Finally, anecdotal accounts indicate political interest in WARN notices. For example, US Senators John McCain (R-AZ), Lindsey Graham (R-SC), and Kelly Ayotte (R-NH) criticized the Obama administration's guidance instructing defense companies and other government contractors not to issue mass layoff notices to their employees under the WARN Act in anticipation of budget cuts scheduled to occur under sequestration on January 2, 2013. They stated,

Today, President Obama put his own reelection ahead of the interests of working Americans and our national security by promising government contractors that their salary and liability costs will be covered at taxpayer expense if they do not follow the law that requires advance warning to employees of jobs that may be lost due to sequestration.^4^

### 2.2. Consequences of Election Uncertainty, Political Uncertainty, and Policy Uncertainty

Political uncertainty affects economic cycles (Bernanke, 1983), equity markets (Baker et al., 2016; Belo et al., 2013; Boutchkova et al., 2012; Brogaard and Detzel, 2015; Pástor and Veronesi, 2012, 2013), and other markets for financial instruments, such as options (Kelly et al., 2016), credit default swaps (Liu and Zhong, 2017), and corporate bonds (Kaviani et al., 2020).

Political uncertainty also has implications for corporate decisions. It affects financing activities, including the timing of initial public offerings and seasoned equity offerings (Chan et al., 2021; Çolak et al., 2017), the level of cash holdings (Duong et al., 2020), and dividend payout policies (Huang et al., 2015) (see Dai and Zhang (2019) for a survey of the literature on political uncertainty and corporate financing decisions). It also affects investment decisions, such as reducing capital expenditures (Gulen and Ion, 2016; Jens, 2017; Julio and Yook, 2012), increasing investments with higher payoffs under uncertainty, e.g., R&D expenditures (Atanassov et al., 2024), decreasing investment efficiency (Drobetz et al., 2018), and reducing M&A activity (Bonaime et al., 2018; Nguyen and Phan, 2017). In addition to financing and investment decisions, political uncertainty affects firms’ production decisions as well (Li and Zhang, 2024).

Political uncertainty affects accounting and financial reporting choices. It influences the frequency of management forecasts and voluntary disclosures (Bird et al., 2023; Nagar et al., 2019), textual features of periodic reports and conference call narratives (Jiang et al., 2022; Mekhaimer et al., 2024), accounting conservatism (Dai and Ngo, 2021), tax avoidance (Nguyen and Nguyen, 2020), and cost stickiness (Lee et al., 2020).

### 2.3. Employment Issues During Election Cycles

Anecdotal and archival evidence indicates that managers, politicians, and employees are aware of increased levels of uncertainty during election periods. Following the election of President Trump in 2016, a Deloitte survey of Chief Financial Officers from many of North America's largest and most influential companies revealed “rapidly rising concerns about US political and policy uncertainty, and also about geopolitical risks and conflicts.”^5^

Several employment-related issues were hotly debated during the 2024 US election cycle, including the future of DEI initiatives, reducing federal workplace oversight, decreasing resources for workplace safety oversight, imposing tariffs on foreign goods, and taxing overtime pay. In a 2020 election speech, President Trump argued that “American workers were smothered by a merciless avalanche of wasteful and expensive and intrusive federal regulation” (Kelly, 2024).

Large layoffs and restructurings have been highlighted in the news during recent elections, such as the closure of a Carrier plant in Indianapolis in 2016, Solyndra's delay of layoff announcements in 2020, the closure of a Stellantis plant in Belvidere in 2023, and the proposed takeover of US Steel by Nippon Steel in 2024. Baccini and Weymouth (2021) examine US presidential elections between 2008 and 2016 and find that white voters tend to vote for Republican challengers in areas with high manufacturing layoffs, while Black voters in these areas tend to vote for Democrats.

## 3. Theoretical Framework and Hypothesis Development

### 3.1 Layoff Decisions Using a Real Options Framework

We use a real options framework, which is commonly used in the literature examining the consequences of political uncertainty, to develop our main prediction. For instance, the model in Bernanke (1983) predicts that the uncertainty of an election increases the value of postponing investments. As noted by Bird et al. (2023: 1031), “A firm faces uncertainty when managers do not have a clear understanding of what may happen. This is most simply understood as stochasticity in expected cash flows, encompassing anything from macro-related demand shocks to doubt over future investment opportunities. If a firm faces increased political uncertainty, its opportunities may change, and investors’ perceptions may change as well.”

In our model, formulated in the Appendix in the e-companion, a risk-neutral representative firm decides to lay off workers and issue a WARN notice now (time 0) or delay this decision until the next period (time 1) when uncertainty concerning demand (time 1) is fully or partially resolved. The timing of a WARN layoff notice is an option on the firm's future earnings and is valuable if the delay helps resolve uncertainty about future economic conditions. Assume the initial (exogenous) level of labor utilized by the firm is $\bar{L}$ while the optimal level of labor, given the (initial) level of uncertainty with no delay, is ${\text{L}}_{0}^{*}<\bar{L}$ . If the firm chooses not to delay layoffs (and WARN notices), then the layoff will be ( $\bar{L}-{\text{L}}_{0}^{*}$ ). If the firm chooses to delay and wait for the resolution of uncertainty, then the optimal level of labor, ${\text{L}}_{1}^{*}(\theta )$ , depends on the realized state of the world, θ , once uncertainty is resolved at time 1. The layoff (if any) would be $\left(\bar{L}-{\text{L}}_{1}^{*}(\theta )\right)$ .

We compare values associated with two alternative decisions at time 0, one where any layoff action or WARN notice occurs at time 0, and the other where (any) layoffs occur at time 1. Note that the firm can make a commitment to an action (layoffs) or delay the action to the next period to take advantage of some resolution of uncertainty. Our hypothesis is that the firm is more likely to delay when uncertainty concerning future outcomes increases. For simplicity, our model assumes that (1) the layoff decision is binary rather than continuous and does not allow for sequential layoffs and (2) demand uncertainty is resolved after the filing of a WARN notice engendered by a layoff action.^6^ We assume that labor demand depends on output demand specified by a horizontal perfectly elastic (competitive) output demand function for time 1, $P=\bar{P}+\theta$ , where θ is a random variable with mean zero and $\bar{P}$ is the expected future output price. Under both strategies, the optimal labor decision depends on output demand at time 1. The formal model is described in detail in the e-companion.

To the extent that future general economic conditions affect the level of output and labor demand and hence the firm's optimal employment of labor, it may be prudent to delay any contemplated labor layoffs when there is high uncertainty about future general economic conditions. Arguably, uncertainty about general economic conditions increases during elections and becomes more pronounced if an election's outcome is less predictable. Thus, it may be rational for a firm to delay issuing a WARN notice in the election period until the outcome is clearer and the uncertainty governing general economic conditions (to the extent that they affect a firm's earnings) is at least partially resolved. However, there is a tradeoff here because delaying any layoffs and WARN notices is costly as additional labor expenses, as well as a civil penalty in addition to back pay and benefits to each affected employee for each day of violation of the notice requirement, are incurred during the waiting period. Changes in the expectations that increase the variance of output (and hence of labor demand) and decrease the mean are likely to increase the value of delaying a layoff and WARN notice. These changes potentially influence managers’ perceived cash flows, the risk-adjusted discount rate, and the expected payoffs of corporate projects.

The conditions described suggest an increase in the standard deviation of demand. According to the comparative statics in the e-companion, the net benefit of delay in labor adjustment (or WARN notices) will increase with a mean-preserving increase in demand uncertainty. Thus, a firm is more likely to delay any labor adjustment as long as the additional labor expenses are not large. As the future becomes more uncertain, the value of waiting for the resolution of uncertainty becomes higher; thus, the firm delays the WARN notice. Note that the analysis proposed above is an explanation in the spirit of real options theory (Dixit and Pindyck, 1994) and suggests that there is value in delaying labor adjustment decisions (and WARN notice issuance) until the resolution of uncertainty (see Trigeorgis and Reuer (2017) for a review of research that appeals to the real options framework for studying firms’ strategic decisions). Our setting involves at least partially irreversible divestment (i.e., plant closures and layoffs), and the prediction of the real options model would apply here; that is, firms find the option to delay valuable and the value of the option increases with uncertainty.

### 3.2 Empirical Prediction

Overall, based on (1) the salience of WARN notices in capturing layoffs, (2) the literature on the effects of election uncertainty, political uncertainty, and policy uncertainty, (3) the evidence linking employment issues to election cycles, and (4) a real options-based framework, we propose the following hypothesis (in alternative form):

*Hypothesis 1:* The likelihood of firms issuing WARN notices decreases during the election quarter and the preceding quarter, but increases in the subsequent quarter.

## 4 State-Level Setting, Data and Sample, and Empirical Design

### 4.1. State-Level Setting

Political uncertainty arising from state-level elections represents a significant shock to firms’ layoff and investment decisions (Jens, 2017). In the United States, there are major elections at the federal and state levels. At the federal level, presidential elections take place every four years, and congressional elections take place every two years, simultaneously with presidential elections or halfway through a president's term (i.e., midterm elections).^7^ At the state level, most states hold gubernatorial elections every four years, except New Hampshire and Vermont, which hold these elections every two years. State legislative elections typically happen every two years, except in Alabama, Louisiana, Maryland, and Mississippi, where they occur every four years. State elections can take place concurrently with presidential or midterm elections or in “off years.” Due to convenience and cost savings, federal and state elections for most states are held at the same time, resulting in periods of election activity primarily in November of even-numbered years. Only five states—Kentucky, Louisiana, Mississippi, New Jersey, and Virginia—hold state elections during odd-numbered “off years.”

Previous studies (e.g., Bird et al., 2023; Jens, 2017) show that political uncertainty associated with state-level elections influences firm behavior because (1) state governments have significant control over economic and labor policies (e.g., taxes, subsidies, budgets, or purchases) and (2) changes in state-level policies affect firms’ perceptions of uncertainty. For example, Alabama enacted HB 56 in 2011, a law that penalizes businesses for hiring undocumented immigrants. The law immediately led to widespread labor shortages and increased uncertainty in the agriculture, hospitality, construction, and fishing industries. Due to the overlapping election calendar in even-numbered years, firms have limited opportunities to mitigate the risks associated with state-level elections (Jens, 2017). Even without this source of political risk, changes in state-level policies could still affect corporate investment decisions (Bernanke, 1983).

The shocks to uncertainty engendered by state-level election cycles offer three practical advantages for our study. First, state-level elections are prescheduled and therefore can be viewed as exogenous events in which political uncertainty arises. Using this setting mitigates the endogeneity problems associated with political uncertainty and financial decisions (Çolak et al., 2017). Second, state-level elections occur much more often than national elections, helping increase variation and enlarge our sample size. Third, since state-level elections occur in different years across states, this allows us to use observations in states without elections as a control group.

Nevertheless, note that we do not make predictions about the exact period in which the layoffs will occur. We do not try to ascertain the timing of the adjustment window, and our main test variable is the election quarter compared to all other quarters, including the same quarter in nonelection years and the quarters surrounding the election period, as our paper speaks to the issue of intertemporal flexibility.

### 4.2. Data and Sample

#### 4.2.1. Data

We manually collected the data on WARN notices from various state Department of Labor websites and supplemented them with machine-readable data from Revelio Labs, a comprehensive source of labor-related data. Because of the limited disclosure guidance provided by the US Department of Labor, each state has developed its own practices for handling of WARN notices. As a result, the availability and accessibility of WARN notices, the duration of the records, and the details of the disclosed information vary by state.

We manually linked each firm that filed a WARN notice to its corresponding GVKEY in Compustat. We complied a dataset of 31,347 WARN notices across 47 states (excluding Arkansas, New Hampshire, and Wyoming, where WARN notices are unavailable), covering 3822 firms for the period from 1994 to 2024.^8^ Unfortunately, individual states do not consistently disclose the reasons for layoffs, such as whether they involve downsizing or a temporary shutdown of a location. Hence, we are unable to collect relevant data to analyze these issues separately.

We obtained the data on state legislative elections from Harvard Dataverse shared by Carl Klarner, which contains state legislative general election returns from 1967 through 2022 for all 50 states. To measure party control, we manually collected data on historical partisan composition of state legislatures from 1992 to 2024 from Ballotpedia. We further gathered the data on gubernatorial elections for the period of 1990 to 2022 from Dave Leip US Election Data. We use several measures to capture political uncertainty, including firm-level political risk (as constructed by Hassan et al., 2019) and state-level economic policy uncertainty (as developed by Baker et al., 2022). We obtained the data for these two measures from the Economic Policy Uncertainty Index website.

We gathered firm-level financial data from Compustat and analyst forecast data from I/B/E/S. We collected the data on state gross domestic product (GDP) and unemployment rates from the US Bureau of Economic Analysis and Bureau of Labor Statistics, respectively. We obtained the unionization data from the Union Membership and Coverage Database (Hirsch et al., 2025). Lastly, to measure a firm's political campaign financing activity, we use the Federal Election Commission (FEC) to Compustat link table shared by Dane Christensen, which links corporate-sponsored political action committees (PACs) from the FEC to Compustat firms (Christensen et al., 2022, 2023).

#### 4.2.2. Sample

 In Table 1, Panel A describes our sample selection procedure. The initial sample starts with 1,228,207 firm-quarter observations (of 31,063 firms) in Compustat universe from 1993 to 2023 after removing duplicates and observations with total assets equal to zero. Following Bird et al. (2023), we use mentions of US states in 10-K filings to construct a time-varying index of a firm's exposure to each state. After merging with the data on 10-K state mentions, our sample contains 7,151,969 firm-state-quarter observations (555,641 firm-quarters of 15,969 firms). We then exclude observations from Arkansas, New Hampshire, and Wyoming, resulting in 6,975,157 firm-state-quarter observations. After removing observations with missing values for major regression variables, our main estimation sample consists of 6,041,649 firm-state-quarter observations, representing 480,353 firm-quarters across 14,106 firms from 1994 to 2022. Our sample size is comparable to that in Bird et al. (2023).

**Table 1.** Sample selection and distribution of WARN notices.

Panel A: Sample selection							
				Firm-state-quarters		Firm-quarters	Firms
Compustat universe from 1993 to 2023 after removing duplicates and observations with total assets equal to zero				N/A		1,228,207	31,063
Merging with 10-K state mentions				7,151,969		555,641	15,969
Excluding observations from Arkansas, New Hampshire, and Wyoming due to the unavailability of WARN notices				6,975,157		555,641	15,969
Requiring nonmissing main regression variables for the sample period of 1994–2022				6,041,649		480,353	14,106
Panel B: Distribution of WARN notices by quarter—relative to the election quarter							
Quarter =	−3	−2	−1	0	1	2	3
Number of observations (*N*)	670,895	690,094	702,156	711,400	606,135	596,033	576,271
*N* with WARN notices	18,435	17,440	15,554	14,950	16,519	14,815	13,295
% With WARN Notices	2.75	2.53	2.22	2.10	2.73	2.49	2.31

*Notes*. This table presents the sample selection procedure (Panel A) and the distribution of WARN notices by quarter**—**relative to the election quarter (Panel B).

### 4.3. Empirical Design

To examine how state election cycles influence the issuance of WARN notices by firms, we estimate the following models:

(1a) $$\begin{array}{rl} WARN_{ist} & =\beta_{0}+\beta_{1}State\;\;Election_{st}+\beta_{\gamma }Controls\\ & \;+Fixed\;\;Effects+\varepsilon_{ist} \end{array}$$

(1b) $$\begin{array}{rl} WARN_{ist} & =\beta_{0}+\beta_{1}Pre-State\;\;Election_{st-1}\\ & \;+\beta_{2}State\;\;Election_{st}+\beta_{3}Post-State\;\;Election_{st+1}\\ & \;+\beta_{\gamma }Controls+Fixed\;\;Effects+\varepsilon_{ist} \end{array}$$

where *i* denotes firm, *s* denotes state, and *t* denotes quarter. Following Bird et al. (2023), we estimate equations (1a) and (1b) using weighted least squares regressions using firm exposures to different states as weights, given that firms may have simultaneous and varying degrees of exposure to elections in multiple states where they operate.^9^ Specifically, the weight, *w*, is the proportion of mentions of state *s* in firm *i*'s 10-K filing for year *t*, calculated as the number of mentions of state *s* divided by the total number of all state mentions in the 10-K. State mentions in 10-K filings provide a reasonable proxy for a firm's operational locations and thus are informative about a firm's exposure to each state in a given year (García and Norli, 2012). This empirical design allows us to exploit variations in firm exposure to elections across states and election cycles.

The dependent variable, *WARN_ist_*, is an indicator variable that equals one if firm *i* issues a WARN notice in state *s* during the current quarter *t*, and zero otherwise. The variables of interest, *PreState Election_st_*_−1_, *State Election_st_*, and *PostState Election_st _*_+ 1_, are indicator variables set to one for the quarter immediately preceding the election (*t*-1) in state *s*, the election quarter *t* in state *s*, and the quarter immediately following the election (*t *+ 1) in state *s*, respectively, and zero otherwise. The indicator variables for the election and surrounding quarters (*t*-1, *t*, *t *+ 1) capture how those periods are different from the average of all other quarters in the sample, making any other “typical” quarter that is not surrounding an election period a baseline. The interquarter analysis (*t*-1, *t*, *t *+ 1) focuses on identifying whether there is any trend within this nine-month period. Overall, our variables of interest and model specification (including preelection, election, and postelection quarters) intend to capture changes in political uncertainty due to the onset of the election followed by resolution of uncertainty once the election is over. In particular, we are interested in examining whether layoffs are less likely to occur in the election quarter and the preceding quarter but are more likely to occur in the subsequent quarter, arguably due to high political uncertainty during state elections.

State elections typically occur in the fourth quarter of the year, and some firms may be reluctant to fire employees in that quarter, which coincides with the holiday season and increased sales activity. To address the confounding effect of seasonality in firms’ layoff decisions, we include an indicator variable, *NonElection Q4_st_*, to control for seasonality. *NonElection Q4_st_* is equal to one for the fourth quarter (*t*) of a nonelection year in state *s*, and zero otherwise. Although the timing of elections in November could be followed by a systematic “January effect” on layoffs for all states, it is difficult to explain why the effect in the January following election years would be different from that in other years for a given firm-state combination. In the presence of seasonality and political uncertainty, we expect that both coefficients on *State Election_st_* and *NonElection Q4_st_* will be negative, and that the coefficient on *State Election_st_* will have a larger magnitude than the coefficient on *NonElection Q4_st_*. That is, firms are even less likely to fire employees and issue WARN notices in the fourth quarter of a state election year compared to a nonstate-election year.

To control for other factors associated with the likelihood of layoffs and the issuance of WARN notices, we incorporate a set of control variables (measured for firm *i* in quarter *t*), including firm size (*SIZE_it_*), leverage (*LEV_it_*), tangibility (*TAN_it_*), financial performance (*ROA_it_*), change in sales (Δ*logSALES_it_*), sales decline (*DEC_it_*), lagged sales decline (*LAGDEC_it_*), and asset intensity (*logAINT_it_*). *SIZE_it_* is measured as the natural logarithm of total assets. *LEV_it_* is computed as total liabilities divided by total assets. *TAN_it_* is defined as the ratio of property, plant, and equipment to total assets. *ROA_it_* is calculated as net income divided by total assets. *logAINT_it_* is calculated as the natural logarithm of the ratio of total assets to sales. Following the literature on cost stickiness (e.g., Lee et al., 2020), we include Δ*logSALES_it_*, calculated as the natural logarithm of the quarterly change in sales, indicator variables for quarterly and lagged quarterly declines in sales (*DEC_it_* and *LAGDEC_it_*), and the interaction term between Δ*logSALES_it_* and *DEC_it_*. The Appendix provides detailed definitions of all variables. In addition to these control variables, we also include year and firm-state fixed effects to control for time trends and time-invariant firm and state characteristics. We adjust standard errors for heteroskedasticity and cluster them at the firm-state level.

## 5. Results

### 5.1. Descriptive Statistics

Table 1, Panel B presents the distribution of WARN notices by quarter, relative to the election quarter. It shows that the likelihood of firms issuing WARN notices is lower in the election quarter (2.1%) and the preceding quarter (2.22%), compared to the following quarter (2.73%). Figure E.1 in the supplemental material in the e-companion provides the corresponding graphical evidence. This descriptive evidence is consistent with our expectation that firms are less likely to issue WARN notices during periods of heightened political uncertainty, such as during or immediately preceding a state election. Instead, they may postpone the issuance of WARN notices until after the election. Table E.1 in the supplemental material in the e-companion presents the distribution of WARN notices by state, which shows that the frequency of WARN notices varies across states.

 Table 2 reports descriptive statistics and Pearson correlations. Panel A compares the differences in control variables between firm-state-quarters with WARN notices and those without. On average, firm-state-quarters with WARN notices (i.e., *WARN *= 1) are larger and more leveraged and have more fixed assets, worse sales performance, and lower asset intensity than those without WARN notices (i.e., *WARN *= 0). Panel B presents descriptive statistics on main regression variables. The mean value of *WARN* indicates that the unconditional likelihood of a WARN notice is 2.3% in our sample. The preelection quarters, election quarters, and postelection quarters account for 11.6%, 11.8%, and 10% of the sample, respectively. Panel C shows the correlations between main regression variables and provides evidence consistent with Panel B of Table 1 and with our expectation that the issuance of WARN notices is lower (higher) in the election and preelection quarters (postelection quarters). The negative correlation between *WARN* and *NonElection Q4* indicates fewer WARN notices in the fourth quarter of a nonelection year.

**Table 2.** Descriptive statistics.

Panel A: Descriptive statistics on control variables							
	*WARN *= 0 (*N *= 5,900,481)			*WARN *= 1 (*N *= 141,168)			
Variable	*M*	Median	SD	*M*		Median	SD
*SIZE*	6.610	6.691	2.253	9.194		9.214	1.772
*LEV*	0.640	0.615	0.365	0.711		0.684	0.242
*TAN*	0.446	0.315	0.424	0.466		0.372	0.381
*ROA*	−0.010	0.006	0.080	0.000		0.008	0.048
Δ*logSALES*	0.076	0.062	0.370	−0.006		0.017	0.268
*DEC*	0.332	0.000	0.471	0.440		0.000	0.496
*LAGDEC*	0.327	0.000	0.469	0.415		0.000	0.493
*logAINT*	1.877	1.632	1.141	1.658		1.483	0.988
Panel B: Descriptive statistics on main regression variables							
Variable (*N *= 6,041,649)		Mean	Median	S.D.		Q1	Q3
*WARN*		0.023	0.000	0.151		0.000	0.000
*PreState Election*		0.116	0.000	0.320		0.000	0.000
*State Election*		0.118	0.000	0.322		0.000	0.000
*PostState Election*		0.100	0.000	0.300		0.000	0.000
*NonElection Q4*		0.136	0.000	0.343		0.000	0.000
*SIZE*		6.671	6.742	2.277		5.173	8.229
*LEV*		0.642	0.617	0.362		0.425	0.799
*TAN*		0.447	0.317	0.423		0.088	0.720
*ROA*		−0.010	0.006	0.080		−0.004	0.017
Δ*logSALES*		0.074	0.061	0.369		−0.042	0.183
*DEC*		0.334	0.000	0.472		0.000	1.000
*LAGDEC*		0.329	0.000	0.470		0.000	1.000
*logAINT*		1.872	1.627	1.138		1.056	2.479
Panel C: Pearson correlations							
Variable	(1)	(2)	(3)	(4)	(5)	(6)	(7)
(1) *WARN*	1.000						
(2) *PreState Election*	**−0**.**003**	1.000					
(3) *State Election*	**−0**.**006**	**−0**.**132**	1.000				
(4) *PostState Election*	**0**.**009**	**−0**.**121**	**−0**.**122**	1.000			
(5) *NonElection Q4*	**−0**.**003**	**−0**.**061**	**−0**.**145**	**−0**.**132**	1.000		
(6) *SIZE*	**0**.**171**	**0**.**010**	**0**.**006**	**0**.**007**	**0**.**001**	1.000	
(7) *LEV*	**0**.**030**	**−0**.**003**	**0**.**005**	0.000	**−0**.**002**	**0**.**028**	1.000
(8) *TAN*	**0**.**007**	**−0**.**012**	**0**.**028**	**−0**.**005**	**0**.**029**	**0**.**067**	**0**.**053**
(9) *ROA*	**0**.**019**	**0**.**011**	**−0**.**020**	**0**.**010**	**−0**.**011**	**0**.**362**	**−0**.**343**
(10) Δ*logSALES*	**−0**.**033**	**0**.**008**	**−0**.**005**	**−0**.**014**	**−0**.**006**	**0**.**014**	**−0**.**083**
(11) *DEC*	**0**.**035**	**−0**.**011**	**0**.**003**	**0**.**013**	**0**.**003**	**−0**.**078**	**0**.**087**
(12) *LAGDEC*	**0**.**028**	**−0**.**007**	**−0**.**008**	**0**.**004**	**0**.**008**	**−0**.**076**	**0**.**091**
(13) *logAINT*	**−0**.**029**	**0**.**005**	**−0**.**002**	0.000	**−0**.**024**	**0**.**237**	**0**.**078**
Variable	(8)	(9)	(10)	(11)	(12)	(13)	
(8) *TAN*	1.000						
(9) *ROA*	**0**.**028**	1.000					
(10) Δ*logSALES*	**−0**.**059**	**0**.**111**	1.000				
(11) *DEC*	**0**.**048**	**−0**.**158**	**−0**.**591**	1.000			
(12) *LAGDEC*	**0**.**051**	**−0**.**132**	**−0**.**400**	**0**.**560**	1.000		
(13) *logAINT*	**−0**.**181**	**−0**.**120**	**−0**.**104**	**0**.**100**	**0**.**083**	1.000	

*Notes*. This table presents the descriptive statistics (Panels A and B) and Pearson correlations (Panel C) of main regression variables. All continuous variables are winsorized at the 1^st^ and 99^th^ percentiles. Variables are defined in the Appendix. In Panel C, bold denotes significance at the 0.10 level or better (two-tailed).

Table E.2 in the supplemental material in the e-companion provides univariate evidence on industry-level sales uncertainty and firm-level earnings uncertainty (proxied by the dispersion of analyst earnings forecasts) around elections. We find that the within-industry standard deviation of changes in sales increases during election periods and preceding quarters and decreases in the postelection period. Furthermore, we find that analysts’ earnings forecast dispersion spikes during election quarters. These findings validate the use of elections as economically meaningful shocks to uncertainty, which can influence firm decision-making.

### 5.2. Main Analyses

 Table 3 reports the effect of state elections on the likelihood of firms issuing WARN notices.^10^ Column (1) shows a negative and significant coefficient on the variable *State Election* (−0.003, *p*- < .01), indicating that firms are less likely to issue mass layoff and plant closing notices during state election periods. Similarly, in Column (2), *State Election* has a negative and significant coefficient (−0.003, *p* < .01). Moreover, *PreState Election* has a negative and significant coefficient (−0.002, *p* < .01) and *PostState Election* has a positive and significant coefficient (0.002, *p* < .01). These results show a trend of fewer WARN notices during and immediately before state elections, followed by an increase in WARN notices after state elections. We find a negative and significant coefficient on *NonElection Q4* (−0.002, *p* < .01), indicating the presence of seasonality in layoffs (and WARN notices). However, the coefficients on *State Election* and *NonElection Q4* have different magnitudes, and the difference is statistically significant in both columns (*p* = .07 in Column (1) and *p* < .001 in Column (2)). This pattern suggests that heightened uncertainty during state elections has an incremental effect on firms’ layoff decisions that further reduces firms’ tendency to lay off employees and issue WARN notices during the fourth quarter of a state election year.

**Table 3.** Likelihood of WARN issuances around state elections.

Dependent variable = *WARN*	(1)	(2)
*PreState Election*		−0.002***
* *		[−7.82]
*State Election*	−0.003***	−0.003***
* *	[−11.47]	[−12.40]
*PostState Election*		0.002***
* *		[6.56]
*NonElection Q4*	−0.002***	−0.002***
* *	[−9.49]	[−7.52]
*SIZE*	0.006***	0.006***
* *	[15.38]	[15.48]
*LEV*	0.007***	0.007***
* *	[13.30]	[13.30]
*TAN*	0.001	0.001
* *	[1.43]	[1.42]
*ROA*	−0.023***	−0.023***
* *	[−16.32]	[−16.27]
Δ*logSALES*	0.005***	0.005***
* *	[14.65]	[14.70]
*DEC*	0.005***	0.005***
* *	[17.90]	[17.86]
*DEC *× Δ*logSALES*	−0.011***	−0.011***
* *	[−15.03]	[−15.11]
*LAGDEC*	0.003***	0.003***
* *	[14.12]	[14.17]
*logAINT*	0.001***	0.001***
* *	[3.86]	[3.72]
*Intercept*	−0.028***	−0.029***
* *	[−12.30]	[−12.35]
*Firm-state fixed effects*	Included	Included
*Year fixed effects*	Included	Included
*N*	6,035,775	6,035,775
Adjusted *R*^2^	0.160	0.160
*p*-value for *State Election *= *NonElection Q4*	.070	<.001

*Notes.* This table presents weighted least squares regression results on the probability of firms issuing WARN notices around state elections. Regressions are weighted by firm exposure to different states mentioned in their 10-K filings. Variables are defined in the Appendix. *t*-statistics in parentheses are calculated using robust standard errors clustered by firm-state. ***, **, and * denote results significant at the 1%, 5%, and 10% levels (two-tailed). The sample sizes here are slightly smaller than those in Panel A of Table 1 because our models include high-dimensional fixed effects that perfectly identify certain observations.

In terms of economic significance, *State Election* is associated with a 13.04% decrease in the likelihood of a WARN notice issuance compared to the average rate of WARN issuances. It means that during a state election quarter, the probability of a firm issuing a WARN notice decreases by about 13.04% compared to the baseline probability of 2.3%. This magnitude represents a meaningful relative reduction in the baseline WARN probability, even though the absolute change in probability associated with state elections seems small (0.3 percentage points), highlighting the impact of state elections on firms’ issuances of WARN notices. The economic magnitude of state elections is comparable to that of other determinants of WARN notices, such as sales declines.

Collectively, these results are consistent with our hypothesis that uncertainty during state elections influences firms’ decisions to announce mass layoffs and plant closures, beyond what can be explained by seasonality. During periods of heightened uncertainty, firms rationally postpone major decisions until the uncertainty diminishes, which leads to the observed delays in issuing WARN notices during state elections, consistent with real options theory.

### 5.3. Cross-Sectional Analyses

In this section, we explore a range of factors that could contribute to the delay in issuing WARN notices during election quarters and perform several cross-sectional analyses to extend our main findings.

#### 5.3.1. Political Risk and Economic Policy Uncertainty

Firms often operate in multiple states and hence face varying degrees of uncertainty from elections in each respective state. State elections introduce uncertainty regarding potential changes in state-level economic policies, taxation, and regulations that may affect firm operations. When firms face higher levels of political risk and economic policy uncertainty (EPU), they are more likely to delay issuing WARN notices until after elections.

 Table 4 reports cross-sectional results conditional on firm-level political risk in Columns (1) and (2) and state-level EPU in Columns (3) and (4). We follow Hassan et al. (2019) and calculate firm-level political risk as the frequency of political bigrams paired with synonyms for “risk” or “uncertainty” divided by the total number of bigrams in quarterly earnings conference call transcripts. This measure quantifies the extent of political risk faced by a firm based on the proportion of dialogue related to political risks during its quarterly earnings conference call. *FIRM_PU* is an indicator variable that equals one if the firm-level political risk is above the sample median, and zero otherwise. Next, we follow Baker et al. (2022) and measure state-level EPU by the proportion of newspaper articles containing terms related to economic conditions, policy, and uncertainty for individual states, capturing the level of uncertainty about state policies. *STATE_EPU* is an indicator variable that equals one if the state-level EPU in the previous quarter is above the sample median, and zero otherwise. Results are similar, albeit slightly weaker, when using the state-level EPU in the current quarter.

**Table 4.** Cross-sectional analyses based on political uncertainty.

	(1)	(2)	(3)	(4)
Dependent variable = *WARN*	*FIRM_PU*	*FIRM_PU*	*STATE_EPU*	*STATE_EPU*
*PreState Election*		−0.003***		−0.001***
* *		[−4.24]		[−3.41]
*PreState Election *× *PU*		−0.003***		−0.002***
* *		[−2.60]		[−3.90]
*State Election*	−0.005***	−0.005***	−0.002***	−0.002***
* *	[−6.15]	[−6.78]	[−5.53]	[−6.50]
*State Election *× *PU*	−0.003***	−0.003***	−0.002***	−0.002***
* *	[−2.58]	[−3.05]	[−3.90]	[−3.87]
*PostState Election*		0.005***		0.001*
* *		[5.48]		[1.73]
*PostState Election *× *PU*		−0.002		0.002***
* *		[−1.40]		[4.10]
*PU*	0.003***	0.003***	−0.000	−0.000
* *	[6.51]	[6.80]	[−1.23]	[−1.52]
*Control variables*	Included	Included	Included	Included
*Firm-state fixed effects*	Included	Included	Included	Included
*Year fixed effects*	Included	Included	Included	Included
*N*	2,571,669	2,571,669	5,264,227	5,264,227
Adjusted *R*^2^	0.191	0.191	0.167	0.167

*Notes.* This table presents weighted least squares regression results on the probability of firms issuing WARN notices around state elections, conditional on political uncertainty. Regressions are weighted by firm exposure to different states mentioned in their 10-K filings. Political uncertainty (*PU*) is measured by: (i) *FIRM_PU*, an indicator variable that equals one if the firm-level political risk, as constructed by Hassan et al. (2019), is above the sample median, and zero otherwise, in Columns (1) and (2); and (ii) *STATE_EPU*, an indicator variable that equals one if the state-level economic policy uncertainty in the previous quarter, as constructed by Baker et al. (2022), is above the sample median, and zero otherwise, in Columns (3) and (4). All other variables are defined in the Appendix. *t*-statistics in parentheses are calculated using robust standard errors clustered by firm-state. ***, **, and * denote results significant at the 1%, 5%, and 10% levels (two-tailed).

We find negative and significant coefficients on *State Election* and *State Election *× *PU* in Columns (1) and (3). Columns (2) and (4) show significantly negative coefficients on *PreState Election* and *State Election* and a significantly positive coefficient on *PostState Election*, identical to the pattern shown in our main findings in Table 3. Furthermore, we find that the interaction terms for *PreState Election *× *PU* and *State Election *× *PU* are negative and significant in Columns (2) and (4), whereas the interaction term for *PostState Election *× *PU* is positive and significant in Column (4). Collectively, these findings suggest that the delay in WARN notices increases when political risk and EPU are high.

#### 5.3.2. Same-Party Control

We explore the influence of the state partisan composition on firms’ issuances of WARN notices during election periods. We propose two measures, *State Control* and *Legislative Control*, based on the political composition of state institutions. Specifically, *State Control* is an indicator variable that equals one if the same party controls the lower house, upper house, and governorship, and zero otherwise. *Legislative Control* is an indicator variable that equals one if the same party controls both the lower and upper houses, and zero otherwise. When the same party controls all political institutions or the legislature in a state, state policies are likely to be more predictable.^11^ Given that unified political control increases the predictability of state policies, it might reduce firms’ perceptions of uncertainty associated with state policies.

 Table 5, Panel A reports cross-sectional results using *State Control* in Columns (1) and (2) and *Legislative Control* in Columns (3) and (4). The main effect of delays in WARN notices during election periods remains. Interestingly, we find a significantly negative coefficient on *State Election *× *Party Control* in all columns, which indicates that the delay in WARN notices increases when the same party holds control over the state or legislature. This finding appears to contradict the argument that policy predictability should reduce uncertainty and potentially lessen the need for such delays. To understand this issue further, in untabulated additional analyses, we find that the negative and significant coefficient on *State Election × Party Control* is observed only in subsamples with high political uncertainty, negative state GDP growth, and high state unemployment rates. In other words, the seemingly counterintuitive results are likely due to the interaction between same-party control, firm-level political uncertainty, and local economic conditions.

**Table 5.** Cross-sectional analyses based on various factors.

Panel A: Same-party control				
	(1)	(2)	(3)	(4)
Dependent variable = *WARN*	*State Control*	*State Control*	*Legislative Control*	*Legislative Control*
*PreState Election*		−0.001***		−0.001***
* *		[−4.35]		[−3.45]
*PreState Election *× *Party Control*		−0.001***		−0.001
* *		[−2.61]		[−1.55]
*State Election*	−0.002***	−0.002***	−0.001***	−0.002***
* *	[−5.89]	[−6.67]	[−3.60]	[−4.31]
*State Election *× *Party Control*	−0.002***	−0.002***	−0.002***	−0.002***
* *	[−4.23]	[−4.36]	[−3.61]	[−3.48]
*PostState Election*		0.001***		0.001**
* *		[4.10]		[2.56]
*PostState Election *× *Party Control*		0.001		0.001
* *		[1.37]		[1.57]
*Party Control*	0.001***	0.001***	0.001***	0.001***
* *	[2.78]	[2.90]	[2.73]	[2.66]
*Control variables*	Included	Included	Included	Included
*Firm-state fixed effects*	Included	Included	Included	Included
*Year fixed effects*	Included	Included	Included	Included
*N*	6,035,775	6,035,775	6,035,775	6,035,775
Adjusted *R*^2^	0.160	0.160	0.160	0.160
Panel B: Political connections				
Dependent variable = *WARN*			(1)	(2)
*PreState Election*				−0.002***
* *				[−7.80]
*PreState Election *× *PAC*				−0.003***
* *				[−2.64]
*State Election*			−0.002***	−0.003***
* *			[−11.54]	[−12.69]
*State Election *× *PAC*			−0.004***	−0.004***
* *			[−3.93]	[−3.95]
*PostState Election*				0.001***
* *				[4.80]
*PostState Election *× *PAC*				0.002*
* *				[1.86]
*PAC*			0.009***	0.009***
* *			[6.97]	[6.94]
*Control variables*	* *	* *	Included	Included
*Firm-state fixed effects*	* *	* *	Included	Included
*Year fixed effects*	* *	* *	Included	Included
*N*			5,699,698	5,699,698
Adjusted *R*^2^			0.168	0.168
Panel C: Local macroeconomic conditions				
	(1)	(2)	(3)	(4)
Dependent variable = *WARN*	*NEG_ΔGDP*	*NEG_ΔGDP*	*UNEMP*	*UNEMP*
*PreState Election*		−0.002***		−0.001***
* *		[−5.61]		[−4.02]
*PreState Election *× *STATE_ECON*		−0.004***		−0.001**
* *		[−4.77]		[−2.52]
*State Election*	−0.002***	−0.003***	−0.002***	−0.002***
* *	[−9.34]	[−9.97]	[−6.74]	[−7.30]
*State Election *× *STATE_ECON*	−0.004***	−0.005***	−0.001***	−0.001***
* *	[−5.27]	[−5.66]	[−2.58]	[−2.91]
*PostState Election*		0.002***		0.001***
* *		[5.88]		[4.03]
*PostState Election *× *STATE_ECON*		0.003***		0.001
* *		[3.00]		[1.22]
*STATE_ECON*	0.000	0.001	0.000	0.001
* *	[1.02]	[1.46]	[1.14]	[1.47]
*Control variables*	Included	Included	Included	Included
*Firm-state fixed effects*	Included	Included	Included	Included
*Year fixed effects*	Included	Included	Included	Included
*N*	5,362,564	5,362,564	6,035,775	6,035,775
Adjusted *R*^2^	0.172	0.172	0.160	0.160
Panel D: Union strength				
	(1)	(2)	(3)	(4)
Dependent variable = *WARN*	*UNION_MEM*	*UNION_MEM*	*UNION_COV*	*UNION_COV*
*PreState Election*		−0.002***		−0.002***
* *		[−7.01]		[−6.44]
*PreState Election *× *UNION*		0.001*		0.001
* *		[1.89]		[1.18]
*State Election*	−0.003***	−0.004***	−0.003***	−0.004***
* *	[−9.96]	[−10.61]	[−9.97]	[−10.56]
*State Election *× *UNION*	0.001**	0.001**	0.001***	0.001**
* *	[2.51]	[2.48]	[2.67]	[2.54]
*PostState Election*		0.002***		0.002***
* *		[5.17]		[5.07]
*PostState Election *× *UNION*		−0.001		−0.000
* *		[−1.06]		[−0.88]
*UNION*	−0.001	−0.001	−0.001	−0.001
* *	[−1.13]	[−1.16]	[−1.06]	[−1.05]
*Control variables*	Included	Included	Included	Included
*Firm-state fixed effects*	Included	Included	Included	Included
*Year fixed effects*	Included	Included	Included	Included
*N*	6,035,775	6,035,775	6,035,775	6,035,775
Adjusted *R*^2^	0.160	0.160	0.160	0.160
Panel E: Firm visibility				
Dependent variable = *WARN*			(1)	(2)
*PreState Election*				−0.001***
* *				[−2.78]
*PreState Election *× *Visibility*				−0.003***
* *				[−6.39]
*State Election*			−0.001***	−0.002***
* *			[−7.72]	[−8.89]
*State Election *× *Visibility*			−0.003***	−0.003***
* *			[−6.14]	[−6.22]
*PostState Election*				0.001**
* *				[2.54]
*PostState Election *× *Visibility*				0.003***
* *				[4.97]

*Visibility*			−0.006***	−0.006***
* *			[−9.87]	[−9.43]
*Control variables*	* *	* *	Included	Included
*Firm-state fixed effects*	* *	* *	Included	Included
*Year fixed effects*	* *	* *	Included	Included
*N*			6,035,775	6,035,775
Adjusted *R*^2^			0.160	0.160

*Notes*. This table presents weighted least squares regression results on the probability of firms issuing WARN notices around state elections, conditional on same-party control (Panel A), political connections (Panel B), local macroeconomic conditions (Panel C), union strength (Panel D), and firm visibility (Panel E). Regressions are weighted by firm exposure to different states mentioned in their 10-K filings. In Panel A, same-party control (*Party Control*) is measured by: (i) *State Control*, an indicator variable that equals one if the same party controls the lower house, upper house, and governorship, and zero otherwise, in Columns (1) and (2); and (ii) *Legislative Control*, an indicator variable that equals one if the same party controls both the lower and upper houses, and zero otherwise, in Columns (3) and (4). In Panel B, *PAC* is an indicator variable that equals one if the firm has reported any political action committee contributions, and zero otherwise. In Panel C, local macroeconomic conditions (*STATE_ECON*) are measured by: (i) *NEG_ΔGDP*, an indicator variable that equals one if the change in the state annual GDP is negative, and zero otherwise, in Columns (1) and (2); and (ii) *UNEMP*, an indicator variable that equals one if the state unemployment rate in the current quarter is above the sample median, and zero otherwise, in Columns (3) and (4). In Panel D, union strength (*UNION*) is measured by: (i) *UNION_MEM*, an indicator variable that equals one if the percentage of employed workers who are union members in a state is above the sample median, and zero otherwise, in Columns (1) and (2); and (ii) *UNION_COV*, an indicator variable that equals one if the percentage of employed workers who are covered by a collective bargaining agreement in a state is above the sample median, and zero otherwise, in Columns (3) and (4). In Panel E, *Visibility* is an indicator variable that equals one if the firm size is above the sample median, and zero otherwise. All other variables are defined in the Appendix. *t*-statistics in parentheses are calculated using robust standard errors clustered by firm-state. ***, **, and * denote results significant at the 1%, 5%, and 10% levels (two-tailed).

#### 5.3.3. Partisanship or Political Connections

Partisanship or political connections may motivate companies to time WARN notices to help their preferred politicians. Bertrand et al. (2018) find that politically connected firms alter their employment decisions to support local politicians during reelection periods by increasing job and plant creation rates and decreasing destruction rates in election years. Similarly, Faccio and Hsu (2017) find that targets of politically connected private equity firms increase employment more during election years and in states with high levels of corruption.

There are mixed views on whether political connections worsen or mitigate agency problems (Wei et al., 2023). It is unclear whether politically connected firms would systematically delay WARN notices until after elections. If, on average, incumbents are favored, firms may delay WARN notices to suppress unfavorable news that can affect reelection chances. Conversely, if challengers are favored, firms may accelerate such notices. To examine the incremental role of partisanship in the timing of firms’ decisions to issue WARN notices, we construct a measure based on whether firms engage in political campaign financing activity (*PAC*) and report cross-sectional results using this measure in Table 5, Panel B. We find significantly negative coefficients on *PreState Election *× *PAC* and *State Election *× *PAC* and a significantly positive coefficient on *PostState Election *× *PAC*. Results in Panel B of Table 5 suggest that the average preference favors incumbents over challengers. Consistent with the partisanship argument, the delay in WARN notices is more pronounced for politically connected firms.

#### 5.3.4. Local Macroeconomic Conditions

During economic downturns, uncertainty may rise due to factors such as pressure on political authorities to change policies or an increased probability of a shift in the ruling party. When uncertainty is higher, the option to delay labor adjustment decisions and their announcements becomes more valuable. In Table 5, Panel C, we examine whether the delay effect is stronger when state elections are coupled with poor local economic conditions, measured by decreases in state GDP (*NEG_ΔGDP*) in Columns (1) and (2) and state unemployment rates (*UNEMP*) in Columns (3) and (4).

We find that the interaction terms for *PreState Election *× *STATE_ECON* and *State Election *× *STATE_ECON* are negative and significant in Columns (2) and (4), whereas the interaction term for *PostState Election *× *STATE_ECON* is positive and significant in Column (2). These findings suggest that local economic conditions incrementally affect firms’ decisions to issue WARN notices during state election periods; specifically, the delay in WARN notices increases under poor economic conditions.

#### 5.3.5. Union Strength

Firms’ decisions to issue WARN notices could be influenced by their mix of stakeholders. Mass layoffs are different from other adverse company news because they have differential impacts on various stakeholders of the firm. Undoubtedly, layoffs are negative news for the affected employees and the unions that represent them; however, they might be positive news for shareholders and creditors owing to potential cost savings from restructuring. Holding layoff decisions constant, firms may be less inclined to delay issuing WARN notices when their employees are highly unionized due to higher costs associated with extended periods between notices and layoffs, such as potential lawsuits, prolonged negotiations, or workforce disruptions.^12^

In Table 5, Panel D, we perform cross-sectional analyses to infer the influence of labor unions, using union membership (*UNION_MEM*) as a proxy in Columns (1) and (2), and coverage by collective bargaining agreements (*UNION_COV*) in Columns (3) and (4). We find a negative and significant coefficient on *State Election* and a positive and significant coefficient on *State Election *× *UNION* in all columns. Collectively, these findings suggest that the delay in WARN notices decreases when unions are strong in a state.

#### 5.3.6. Firm Visibility

Our paper builds on literature that links increased uncertainty during election cycles, as well as variations in political uncertainty and policy uncertainty, to investment and other corporate decisions. Research in this literature typically argues that “future government policy is largely unpredictable and changes over time with political turnover and shifting policy preferences” (Atanassov et al., 2024: 2937). Moreover, several papers apply a real options theoretical framework based on Bernanke (1983) and others, where, given nonconvex adjustment costs, uncertainty creates a valuable option to delay major decisions.

The visibility or size of a firm is ambiguously related to uncertainty. On the one hand, comparatively large firms may be able to diversify state-level political risks by operating in multiple states or relocating their headquarters or manufacturing sites. For example, Tesla moved its headquarters from California to Texas in 2021, citing reasons like stronger labor laws, higher living costs, and taxes in California versus cheaper labor and less stringent regulations in Texas. On the other hand, large firms’ plant closing and mass layoff decisions may face higher scrutiny during election periods. An example is the Stellantis plant closure in 2023, where several observers claimed that the company was awaiting the outcome of the 2024 elections before considering reopening the plant. Hence, the incremental role of visibility over uncertainty is an open question.

We investigate whether visibility, proxied by firm size, is associated with firms’ decisions to issue WARN notices during election periods. The results are presented in Table 5, Panel E. We find significantly negative coefficients on *PreState Election *× *Visibility* and *State Election *× *Visibility* and a significantly positive coefficient on *PostState Election *× *Visibility*. These findings suggest that the delay in WARN notices increases when firms are larger and more visible. However, we caution that firm size captures not only visibility but also other characteristics related to firm size, such as the ability to pledge collateral for debt financing and bankruptcy rates (e.g., Almeida and Campello, 2007; Rampini and Viswanathan, 2013).

### 5.4. Additional Analyses

#### 5.4.1. State-Level Election Cycles That Involve Governor Appointments

There are two major elections at the state level, gubernatorial and state legislative elections, and these two often overlap every four years. Our study focuses on state legislative elections because state legislatures have authority over laws, regulations, and budgets. Gubernatorial elections, while important, typically focus on broader state-wide issues and may not result in immediate policy changes that directly affect firms’ operations. To control for the impact of gubernatorial elections, we incorporate indicator variables for these elections into our models and report the results in Table E.3 in the supplemental material, Panel A in the e-companion. Our main results hold for the state election variables, while the gubernatorial election variables are insignificant, suggesting that uncertainties associated with state legislative elections appear to have a more prominent effect on firms’ layoff announcements during election periods than gubernatorial elections.

#### 5.4.2. Size of Layoffs

In our main analyses, we focus on whether firms issue WARN notices during state elections. As an additional test, we use an alternative dependent variable that measures the size of layoffs (*Layoffs*) and present the results in Panel B of Table E.3 in the supplemental material in the e-companion. *Layoffs* is measured by the number of employees laid off as disclosed in WARN notices, and is set to zero for observations with missing information in WARN notices or those without WARN notices. Specifically, we use the natural logarithm of one plus the number of affected employees disclosed in a WARN notice as the dependent variable in our regression models. The results are consistent with those reported in Table 3 using *WARN* as the dependent variable, suggesting that state election cycles affect both the issuance of WARN notices and the number of employees laid off.

#### 5.4.3. Ex-Post Financial Performance

If the delay in employee dismissals is due to a rational, value-enhancing decision to wait, we would expect no significant deterioration in financial performance for delayers compared to nondelayers. We define delayers (nondelayers) as firms issuing WARN notices one quarter after state elections (during election quarters). Table E.4 in the supplemental material in the e-companion compares ROA and profitability of delayers and nondelayers over the eight quarters starting from the election quarter. The horizon examined in Table E.4 in the supplemental material extends only up to seven quarters after the election (i.e., quarter *t *+ 7) because most states follow a two-year election cycle, making quarter *t *+ 8 the election quarter in the next cycle. Delayers appear to have better financial performance than nondelayers in the election quarter and the following quarter, suggesting that the decision to delay workforce adjustments may be associated with better financial outcomes in the short run. However, from beyond one year after the election (i.e., from quarter *t *+ 5), there is no clear evidence on whether delayers perform better than nondelayers. Overall, we do not see a substantial deterioration in delayers’ financial performance compared to nondelayers.

#### 5.4.4. Layoffs Covered by the Media

Firms can conduct mass layoffs with or without WARN notices, but they will face penalties if they do not comply with the requirement under the WARN Act to give timely notice to affected employees. Arguably, seeking to avoid attention from mass layoffs during election periods, firms may opt to pay penalties instead of filing WARN notices. We perform an exploratory analysis to provide descriptive evidence on whether the patterns of layoffs with versus without WARN notices are different around elections. To identify firms conducting layoffs without WARN notices, we obtain the data on layoff news from RavenPack. We then merge them with our WARN data, allowing us to distinguish layoffs with and without WARN notices.^13^ Table E.5 in the supplemental material in the e-companion reports the distribution of layoffs without WARN notices by quarter, compared to the election quarter, and Figure E.2 in the supplemental material in the e-companion presents the corresponding graphic evidence. Unlike Figure E.1 in the supplemental material, which shows that WARN notices increase in the quarter immediately after elections, Figure E.2 in the supplemental material shows that the peak of layoffs without WARN notices occurs during the election quarter. Unreported *t*-tests indicate that the likelihood of layoffs without WARN notices during the election quarter is statistically different from that in the quarter immediately preceding or following the election (*p* < .001). These findings suggest that some firms might lay off employees without WARN notices during the election quarter to avoid attention. However, we urge caution in interpreting these results due to their exploratory and descriptive nature.

## 6. Conclusion

We study the relation between political uncertainty during state-level election cycles and the timing of employee dismissal and plant closure notices by US firms under the WARN Act of 1988. Our main results show that the likelihood of WARN notices is significantly lower in the election quarter and the quarter immediately before the election but is significantly higher in the quarter immediately after the election. This pattern aligns with predictions from the real options framework. Our cross-sectional analyses show a range of factors that can mitigate or accentuate firms’ delays in WARN notices during election periods.

Our empirical analyses come with some caveats. We acknowledge that there are other factors that could influence the issuance of WARN notices, which are not examined in this study. For example, there could be threats to management control from capital, labor, and product markets (Berger and Ofek, 1995; Denis and Denis, 1995; Denis and Shome, 2005; Weisbach, 1988), as well as a negative impact on manager reputation (Flanagan and O'Shaughnessy, 2005). Managers may want to avoid unpleasant feelings from firing employees (Grunberg et al., 2006). However, the foregoing explanations are likely to hold generally and are unlikely to vary systematically with election cycles. Furthermore, despite empirical attempts, we cannot completely remove the effect of seasonality to the extent that it overlaps with election cycles. Additionally, as mentioned, political uncertainty and partisanship effects reinforce each other, where firms have incentives to influence election outcomes in a way to support their political allies through strategic decisions around election cycles. We also acknowledge that it is difficult to pinpoint the period when uncertainty arising from an election reaches a peak. Each firm has its own planning and decision-making horizon. Corporate layoffs and investments often take time to implement, making it difficult for firms to make rapid changes to their plans. Nevertheless, our analyses explore variations in uncertainty around elections and focus on changes in uncertainty over time.

An important unanswered question concerns the social welfare implication of corporate restructuring decisions (including layoffs) during election periods. While the focus of this paper is on the timing of WARN notices in the context of election cycles and on potential agency problems within the firm, the importance of the issue we have addressed is broader. Social welfare and public policy issues stem from the following questions: What are the impacts of WARN notices on the length of the subsequent unemployment, on postunemployment earnings, and on the caliber of subsequent jobs? More broadly, public policy questions pertain to the impact of WARN notices on the characteristics of the labor market, including employment and the labor turnover rate. A further question related to our paper is the following: Do our documented WARN notice timing effects (in the election cycle) alter the patterns of the impact of WARN notices on the levels of subsequent unemployment and postunemployment earnings? We leave the examination of these issues to future research.

## Supplemental Material

sj-docx-1-pao-10.1177_10591478251331149 - Supplemental material for Political Uncertainty and the Timing of Mass Layoffs Supplemental material, sj-docx-1-pao-10.1177_10591478251331149 for Political Uncertainty and the Timing of Mass Layoffs by Varouj Aivazian, Tzu-Ting Chiu, Miguel Minutti-Meza and Dushyantkumar Vyas in Production and Operations Management

## Appendix. Variable Definitions

**Table:**

Variable	Definition	Data source
Dependent variable		
*WARN*	An indicator variable that equals one if the firm issues a WARN notice during the current quarter, and zero otherwise.	State Department of Labor websites; Revelio Labs
Variables of interest		
*State Election*	An indicator variable that equals one for the state election quarter, and zero otherwise.	State Legislative Election Returns on Harvard Dataverse
*PreState Election*	An indicator variable that equals one for the quarter immediately preceding the state election, and zero otherwise.	State Legislative Election Returns on Harvard Dataverse
*PostState Election*	An indicator variable that equals one for the quarter immediately following the state election, and zero otherwise.	State Legislative Election Returns on Harvard Dataverse
Control variables		
*NonElection Q4*	An indicator variable that equals one for the fourth quarter of a nonstate-election year, and zero otherwise.	State Legislative Election Returns on Harvard Dataverse
*SIZE*	The natural logarithm of total assets.	Compustat
*LEV*	Total liabilities divided by total assets.	Compustat

*TAN*	Property, plant, and equipment scaled by total assets.	Compustat
*ROA*	Net income divided by total assets.	Compustat
Δ*logSALES*	The natural logarithm of the change in sales in the current quarter relative to the same quarter of the prior year.	Compustat
*DEC*	An indicator variable that equals one if sales decrease during the current quarter, and zero otherwise.	Compustat
*LAGDEC*	An indicator variable that equals one if sales decreased during the previous quarter, and zero otherwise.	Compustat
*logAINT*	The natural logarithm of the ratio of total assets to sales.	Compustat
Cross-sectional variables		
Political uncertainty (*PU*) measures		
*FIRM_PU*	An indicator variable that equals one if the firm-level political risk is above the sample median, and zero otherwise. Following Hassan et al. (2019), the firm-level political risk is calculated as the frequency of political bigrams paired with synonyms for “risk” or “uncertainty” divided by the total number of bigrams in quarterly earnings conference call transcripts.	Economic Policy Uncertainty Index website
*STATE_EPU*	An indicator variable that equals one if the state-level economic policy uncertainty (EPU) in the previous quarter is above the sample median, and zero otherwise. Following Baker et al. (2022), the state-level EPU index is measured by the proportion of newspaper articles containing terms about the economy, uncertainty, and policy, reflecting the level of uncertainty within a state that arises from state and local policy issues.	Economic Policy Uncertainty Index website
Party control (*Party Control*) measures		
*State Control*	An indicator variable that equals one if the same party controls the lower house, upper house, and governorship, and zero otherwise.	Ballotpedia website
*Legislative Control*	An indicator variable that equals one if the same party controls both the lower and upper houses, and zero otherwise.	Ballotpedia website
Political connection measure		
*PAC*	An indicator variable that equals one if the firm has reported any political action committee contributions, and zero otherwise.	Dane Christensen's website
Local macroeconomic condition (*STATE_ECON*) measures		
*NEG_ΔGDP*	An indicator variable that equals one if the change in the state annual GDP is negative, and zero otherwise.	US Bureau of Economic Analysis
*UNEMP*	An indicator variable that equals one if the state unemployment rate in the current quarter is above the sample median, and zero otherwise.	US Bureau of Labor Statistics
Union strength (*UNION*) measures		
*UNION_MEM*	An indicator variable that equals one if the percentage of employed workers who are union members in a state is above the sample median, and zero otherwise.	Union Membership and Coverage Database
*UNION_COV*	An indicator variable that equals one if the percentage of employed workers who are covered by a collective bargaining agreement in a state is above the sample median, and zero otherwise.	Union Membership and Coverage Database
Firm visibility measure		
*Visibility*	An indicator variable that equals one if the firm size is above the sample median, and zero otherwise.	Compustat
Additional variables		
*Industry-Level Sales Uncertainty*	The standard deviation of Δ*logSALES* within an industry, where Δ*logSALES* is calculated as the natural logarithm of the change in sales in the current quarter relative to the same quarter of the prior year.	Compustat

*Firm-Level Forecast Dispersion*	The standard deviation of analysts’ quarterly earnings forecasts scaled by the mean forecast.	I/B/E/S
*Gubernatorial Election*	An indicator variable that equals one for the gubernatorial election quarter, and zero otherwise.	Dave Leip US Election Data
*PreGubernatorial Election*	An indicator variable that equals one for the quarter immediately preceding the gubernatorial election, and zero otherwise.	Dave Leip US Election Data
*PostGubernatorial Election*	An indicator variable that equals one for the quarter immediately following the gubernatorial election, and zero otherwise.	Dave Leip US Election Data
*Layoffs*	The number of employees laid off as disclosed in WARN notices, which is set to zero for observations with missing information in WARN notices or those without WARN notices.	State Department of Labor websites; Revelio Labs
*Profitability*	Earnings before interest, taxes, depreciation, and amortization divided by sales.	Compustat

## Notes

1. Reflecting the complexity associated with downsizing decisions, the findings reported in the prior literature are inconclusive. Actions that change the structure of a firm’s assets and reduce its employee base may increase shareholder wealth (Berger and Ofek, 1995; Statman and Sepe, 1989), but employee dismissals may also signal low expected profitability and impending financial distress. Chatrath et al. (1995) and Palmon et al. (1997) document a positive market reaction to employee dismissals, while Worrell et al. (1991), Hallock (1998), and Hillier et al. (2007) show a negative market reaction. Kashefi and McKee (2002) find a positive reaction for firms with proactive announcements (i.e., those that are part of a strategic restructuring plan) but a negative reaction for firms with reactive announcements (i.e., those that are a direct response to financial distress). The effects of downsizing (and of the WARN legislation in general) are also found to vary with firm size (Alexander and Spivey, 1997).

2. https://www.nytimes.com/interactive/2020/11/03/us/elections/results-florida-amendment-2-raise-minimum-wage.html(accessed 3 March 2025).

3. If the plant closing or layoff event is material to a firm’s business operations, the notice to state labor authorities and employees should trigger a disclosure through an SEC filing (Form 8-K). Note that the issuance of a WARN notice is likely to prompt an 8-K filing, not the reverse. Thus, WARN notices serve as a way to identify substantial changes in a company’s workforce.

4. House Hearing, 113 Congress from the U.S. Government Publishing Office https://www.govinfo.gov/content/pkg/CHRG-113hhrg78696/html/CHRG-113hhrg78696.htm (accessed 3 March 2025).

5. See page 8 of the report at https://www2.deloitte.com/us/en/pages/finance/articles/cfos-signals-survey-growing-concerns-about-political-and-policy-uncertainty.html (accessed 3 March 2025).

6. While these assumptions may be not be fully realistic (a partial labor adjustment process would indeed be more realistic), we believe that they do not unduly constrain the generality of our central hypothesis that the likelihood of delays in layoffs and WARN notices increases with uncertainty.

7. The US Senate has 100 members, elected for a 6-year term in dual-seat constituencies (two from each state). Approximately one-third of the Senate seats are up for election every two years. The US House of Representatives has 435 members, elected for a two-year term in single-seat constituencies. All House seats are up for election every two years.

8. Relatively few states offer historical WARN notice data dating back to the mid-nineties. In addition, WARN notices are unavailable for Arkansas, New Hampshire, and Wyoming because (1) Arkansas and Wyoming do not publish WARN notices due to confidentiality requirements, and (2) New Hampshire does not make WARN notices freely available online, which can only be obtained via a public information request.

9. We use weighted least squares regressions for estimation because it is not possible to use conditional logistic regressions when the model includes high-dimensional fixed effects and applies weights proportional to state exposure.

10. Note that the sample sizes in our regression analyses are slightly smaller than those in Panel A of Table 1 due to the inclusion of high-dimensional fixed effects in our models, which perfectly identify certain observations.

11. Alternatively, we construct measures based on whether the same party controls the lower or upper house for 8 or 12 consecutive years. However, we do not find any incremental effect from these measures of multiple cycles with the same party in power (in either the lower or upper house) on delays in WARN notices during election periods, highlighting the role of a unified partisan composition across political institutions in a state.

12. We acknowledge that unionization increases the adjustment costs of layoffs, which would directly affect firms’ hiring and firing decisions. Moreover, instead of timely layoff notices, unions probably aim to preserve jobs for their members.

13. Identifying layoffs using the news data from RavenPack may be biased towards large firms because large firms are more likely to be covered by the media. However, as explained in Section 5.3.6, large firms tend to be more visible and receive more attention. Therefore, if firms strategically choose to go under radar by not filing WARN notices during election periods, this effect would be more pronounced in large and visible firms.
