# Supplementary material for: Political Uncertainty and the Timing of Mass Layoffs
Source: Prod Oper Manag. 2025 Apr 2;34(10):3193–213. doi: 10.1177/10591478251331149 (PMC13046240; doi:10.1177/10591478251331149)
Supplement: sj-docx-1-pao-10.1177_10591478251331149 - Supplemental material for Political Uncertainty and the Timing of Mass Layoffs [file sj-docx-1-pao-10.1177_10591478251331149.docx]

**E-Companion**

**for**

**“****Political Uncertainty and the Timing of Mass Layoffs”**

by

Varouj Aivazian, University of Toronto

Tzu-Ting Chiu, NHH Norwegian School of Economics

Miguel Minutti-Meza, University of Miami

Dushyantkumar Vyas, University of Toronto

This e-companion provides supplementary materials to the manuscript titled “Political Uncertainty and the Timing of Mass Layoffs.”

Supplementary materials included in the e-companion are listed below:

Online appendix: Theoretical model and proof for the framework presented in Section 3

Figure E.1: Graph of the distribution of WARN notices around the election quarter

Figure E.2: Graph of the distribution of layoffs without WARN notices around the election quarter

Table E.1: Distribution of WARN notices by state

Table E.2: Sales and earnings uncertainty around the election quarter

Table E.3: Additional analyses

Table E.4: Comparison of financial performance between delayers and non-delayers

Table E.5: Distribution of layoffs without WARN notices by quarter—relative to the election quarter

**Online appendix: Theoretical model and proof for the framework presented in Section 3**

***A1.1 The model***

We develop a two-period model, time 0 (*ex ante*) and time 1 (*ex post*). A representative competitive firm carries out production to satisfy sales that only occur at time 1 (*ex post*). The firm is risk neutral and makes its labor employment decisions and adjustments either *ex ante* or *ex post*. The firm faces ex-post demand that is uncertain (at time 0), and uncertainty affects the output price.

The ex-post profit function is: $\pi$ =$\left\{ \begin{matrix} \left( \bar{P}+\theta\right)\cdot2A\left( L \right)-2wL , with probability: \gamma\\ \left( \bar{P}-\theta\right)\cdot2A\left( L \right)-2wL , with probability: 1- \gamma\end{matrix} \right.$.

A(L) is the firm’s overall production function; w > 0 is the wage rate per period. L denotes labor employed; $\bar{L}$ is exogenous and denotes the initial level of labor employed. Note that $\pm\theta$ is the realization of a demand shock on output price, $0<\theta<\bar{P}$, and $\gamma$ is the probability of a positive demand shock, i.e.,$\bar{P}+\theta$. We assume that the sale of goods occurs *ex post* while production can take place both *ex ante* and *ex post*. Without loss of generality, we assume a risk-free rate of return: r_f_ = 0. Note that the ex-post profit function assumes that L is constant across the two periods. This assumption will be relaxed in Scenario 2.

***Firm’s optimal labor decision***

We consider two scenarios:

*Scenario 1: Make labor adjustment at time 0*

The representative firm’s production targets output demand at time 1 that is uncertain at time 0. Uncertainty is fully resolved at time 1. The firm can choose to adjust (or lay off) workers and issue a WARN notice *ex ante* at time 0 or wait until the resolution of demand uncertainty *ex post* at time 1. By laying off workers at time 0, the firm saves labor wage costs.

The firm faces the following profit function:

$\max_{L} \left\{ \left( \left( \bar{P}+\theta\right) \gamma+\left( \bar{P}-\theta\right) \left( 1-\gamma\right) \right)\cdot2A\left( L \right)-2wL \right\}$ (1)

Denoting optimal labor from Eq. (1) as L_0_*, the resulting optimal firm value at time 0 is:

$V_{0}$= $\left( \left( \bar{P}+\theta\right) \gamma+\left( \bar{P}-\theta\right) \left( 1-\gamma\right) \right)\cdot2A\left( L_{0}^{*} \right)-2wL_{0}^{*}$ (1a)

If $\bar{L}>L_{0}^{*}$, then optimal ex-ante layoff (and WARN issuance) is $\bar{L}-L_{0}^{*}$.

*Scenario 2: Make labor adjustment at time 1*

As shown in Scenario 1, the firm may lay off workers and issue a WARN notice at time 0 based on its expected output demand. However, in view of the approaching election, it may choose to delay that decision. The firm now faces the following profit function:

$$\max_{L_{1H}} \left\{ \left( \left( \bar{P}+\theta\right)\left( A\left( \bar{L} \right)+A\left( L_{1H} \right) \right)-w\left( \bar{L}+L_{1H} \right) \right)\gamma\right\}$$

$+\max_{L_{1L}} \left\{ \left( \left( \bar{P}-\theta\right)\left( A\left( \bar{L} \right)+A\left( L_{1L} \right) \right)-w\left( \bar{L}+L_{1L} \right) \right)\left( 1-\gamma\right) \right\}$ (2)

Labor employed is now state dependent: L_1_* = $\left\{ \begin{matrix} L_{1H} for state: \left( \bar{P}+\theta\right) \\ L_{1L} for state: \left( \bar{P}-\theta\right) \end{matrix} \right.$, and firm value at time 0 is based on optimal labor adjustment *ex post*:

$V_{1}$ $=\left( \left( \bar{P}+\theta\right)\left( A\left( \bar{L} \right)+A\left( L_{1H}^{*} \right) \right)-wL_{1H}^{*} \right)\gamma$

$+\left( \left( \bar{P}-\theta\right)\left( A\left( \bar{L} \right)+A\left( L_{1L}^{*} \right) \right)-wL_{1L}^{*} \right)\left( 1-\gamma\right)-w\bar{L}$ (2a)

The labor adjustment or layoff (if any) is also state dependent: $\bar{L}-L_{1H}^{*}$ or $\bar{L}-L_{1L}^{*}$. It occurs *ex post* (at time 1). If $\bar{L}>L_{1}^{*}$, the firm lays off workers. If$\bar{L}<L_{1}^{*}$, the firm hires more workers. Consider the difference between (optimal) firm values for the two alternative labor adjustment actions above (*ex ante* versus *ex post*):

$DV=V_{1}-V_{0}$ (3)

We get the following expression:

$$DV=\left( \left( \bar{P}+\theta\right)\left( A\left( \bar{L} \right)+A\left( L_{1H}^{*} \right) \right)-wL_{1H}^{*} \right)\gamma$$

$$+\left( \left( \bar{P}-\theta\right)\left( A\left( \bar{L} \right)+A\left( L_{1L}^{*} \right) \right)-wL_{1L}^{*} \right)\left( 1-\gamma\right)-w\bar{L}$$

$-\left( \left( \left( \bar{P}+\theta\right) \gamma+\left( \bar{P}-\theta\right) \left( 1-\gamma\right) \right)\cdot2A\left( L_{0}^{*} \right)-2wL_{0}^{*} \right)$ (4)

$DV=\left( \begin{matrix} \left( \begin{matrix} \left( \bar{P}+\theta\right)\left( A\left( \bar{L} \right)+A\left( L_{1H}^{*} \right)-2A\left( L_{0}^{*} \right) \right) \\ -w\left( L_{1H}^{*}-L_{0}^{*} \right) \end{matrix} \right)\gamma+ \\ \left( \begin{matrix} \left( \bar{P}-\theta\right)\left( A\left( \bar{L} \right)+A\left( L_{1L}^{*} \right)-2A\left( L_{0}^{*} \right) \right) \\ -w\left( L_{1L}^{*}-L_{0}^{*} \right) \end{matrix} \right)\left( 1-\gamma\right) \end{matrix} \right)-w\left( \bar{L}-L_{0}^{*} \right)$ (4a)

***Cost of delaying labor adjustment (layoffs)***

***Option value of delaying labor adjustment (layoffs)***

The first term in (4a) is the option value of delaying labor adjustment (layoffs), while the second term stands for the cost of delaying labor adjustment. If $DV>0$, the firm will optimally choose to delay labor adjustment. Note that *DV* is the net benefit from delaying labor adjustment. The first term is the option value of delay, and the second term is the cost of delay, which is the labor compensation (wage bill) that could be avoided by laying off workers earlier.

***A1.2 Illustration: Cobb-Douglas production function***

We solve for the optimal labor adjustment decision and for the comparative statistics of changes in that decision when there is a mean-preserving increase in uncertainty.

*Parameter definition*:

Probability distribution: $\left\{ \begin{aligned} \bar{P}+\theta with probability of 0.5; \\ \bar{P}-\theta with probability of 0.5. \end{aligned} \right.$

This implies that an increase in $\theta$ is a mean-preserving change in uncertainty.

$A\left( L \right)$ =$aL^{b}$ is the production function per period.

Parameters *a,* $\bar{P},$ $\theta$ are greater than zero; *b* is between 0 and 1; $\bar{P}>\theta$ to ensure a positive price in all cases.

Optimal labor with the level of employment adjusted *ex ante*:

$\max_{L}\left\{ 0.5\left( \bar{P}+\theta\right)\cdot2aL^{b}+0.5\left( \bar{P}-\theta\right)\cdot2aL^{b}-2wL \right\}$ (5)

$L_{0}^{*}=\left( ab\bar{P}/w \right)^{1/(1-b)}$ (5a), assume $\bar{L}$ > $L_{0}^{*}$.

Optimal labor with the level of employment adjusted *ex post*:

$0.5\max_{L}\left\{ \left( \bar{P}+\theta\right)\left( a\left( \bar{L}^{b} \right)+a\left( L^{b} \right) \right)-w\bar{L}-wL \right\}+0.5\max_{L}\left\{ \left( \bar{P}-\theta\right)\left( a\left( \bar{L}^{b} \right)+a\left( L^{b} \right) \right)- w\bar{L}-wL \right\}$ (6)

$L_{11}^{*}=\left( ab\left( \bar{P}+\theta\right)/w \right)^{1/(1-b)}$ (6a)

$L_{12}^{*}=\left( ab\left( \bar{P}-\theta\right)/w \right)^{1/(1-b)}$ (6b)

Comparative statics: $\frac{\partial DV}{\partial\theta}>0 \forall\theta$. Specifically, it shows that, within this framework (for binomial shocks), the net benefit from delaying labor adjustment (layoffs) increases with uncertainty and that the option value of delay is positive.

**Figure E.1**

**Graph of the distribution of WARN notices around the election quarter**

This figure presents the graph of the distribution of WARN notices around the election quarter. Quarter 0 refers to the election quarter, Quarter -1 (1) refers to one quarter before (after) the election quarter, and so on.


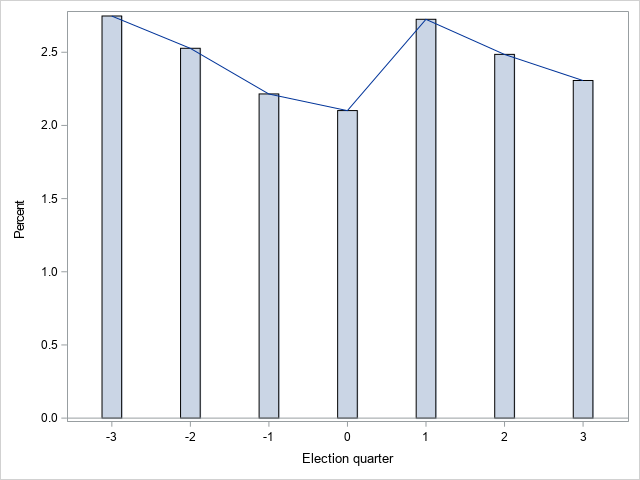


**Figure E.2**

**Graph of the distribution of layoffs without WARN notices around the election quarter**

This figure presents the graph of the distribution of layoffs without WARN notices around the election quarter. Quarter 0 refers to the election quarter, Quarter -1 (1) refers to one quarter before (after) the election quarter, and so on.


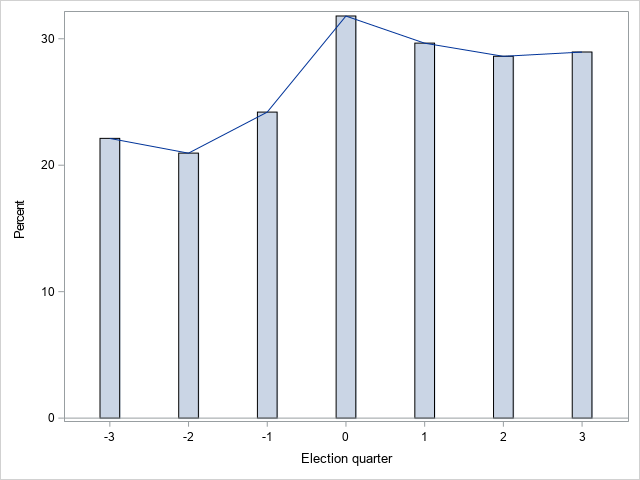


| **Table E.1** | | | | |
| --- | --- | --- | --- | --- |
| **Distribution of WARN notices by state** | | | | |
| State | # of firms | # of observations | # of observations with WARN notices | % of observations with WARN notices |
| Alaska | 1,791 | 29,069 | 1,150 | 3.96 |
| Alabama | 4,153 | 88,359 | 2,636 | 2.98 |
| Arizona | 5,197 | 111,647 | 2,944 | 2.64 |
| California | 11,475 | 312,991 | 6,176 | 1.97 |
| Colorado | 7,784 | 154,974 | 3,239 | 2.09 |
| Connecticut | 6,044 | 115,736 | 2,715 | 2.35 |
| Delaware | 12,524 | 378,080 | 6,694 | 1.77 |
| Florida | 8,158 | 186,358 | 4,345 | 2.33 |
| Georgia | 6,624 | 148,079 | 3,805 | 2.57 |
| Hawaii | 2,107 | 37,815 | 1,298 | 3.43 |
| Iowa | 3,254 | 64,841 | 1,782 | 2.75 |
| Idaho | 5,232 | 61,760 | 1,612 | 2.61 |
| Illinois | 8,443 | 191,763 | 4,360 | 2.27 |
| Indiana | 12,271 | 348,405 | 5,620 | 1.61 |
| Kansas | 4,037 | 88,857 | 2,422 | 2.73 |
| Kentucky | 3,482 | 81,752 | 2,431 | 2.97 |
| Louisiana | 4,377 | 100,286 | 2,314 | 2.31 |
| Massachusetts | 7,887 | 160,405 | 3,318 | 2.07 |
| Maryland | 6,898 | 150,802 | 3,782 | 2.51 |
| Maine | 2,258 | 42,311 | 1,267 | 2.99 |
| Michigan | 6,055 | 129,189 | 3,282 | 2.54 |
| Minnesota | 4,946 | 104,305 | 2,679 | 2.57 |
| Missouri | 4,599 | 100,326 | 2,782 | 2.77 |
| Mississippi | 4,092 | 86,969 | 2,215 | 2.55 |
| Montana | 1,986 | 39,422 | 1,125 | 2.85 |
| North Carolina | 5,987 | 133,362 | 3,538 | 2.65 |
| North Dakota | 1,742 | 32,686 | 906 | 2.77 |
| Nebraska | 4,151 | 87,136 | 2,103 | 2.41 |
| New Jersey | 7,326 | 159,951 | 3,626 | 2.27 |
| New Mexico | 3,600 | 76,621 | 2,154 | 2.81 |
| Nevada | 5,652 | 123,393 | 2,974 | 2.41 |
| New York | 12,389 | 355,804 | 6,980 | 1.96 |
| Ohio | 6,270 | 152,031 | 4,147 | 2.73 |
| Oklahoma | 3,782 | 85,156 | 2,321 | 2.73 |
| Oregon | 8,367 | 138,535 | 2,481 | 1.79 |
| Pennsylvania | 8,306 | 189,564 | 4,272 | 2.25 |
| Rhode Island | 2,431 | 44,215 | 1,368 | 3.09 |
| South Carolina | 3,912 | 84,964 | 2,532 | 2.98 |
| South Dakota | 2,008 | 36,172 | 1,030 | 2.85 |
| Tennessee | 4,796 | 113,684 | 3,107 | 2.73 |
| Texas | 9,829 | 253,677 | 5,391 | 2.13 |
| Utah | 3,947 | 79,651 | 2,038 | 2.56 |
| Virginia | 7,465 | 160,888 | 3,758 | 2.34 |
| Vermont | 2,044 | 40,099 | 1,565 | 3.90 |
| Washington | 9,611 | 230,946 | 4,638 | 2.01 |
| Wisconsin | 4,583 | 97,046 | 2,763 | 2.85 |
| West Virginia | 2,297 | 51,567 | 1,483 | 2.88 |
| This table presents the distribution of WARN notices by state. | | | | |

| **Table E.2** | | | | | | |
| --- | --- | --- | --- | --- | --- | --- |
| **Sales and earnings uncertainty around the election quarter** | | | | | | |
| Variable |  |  | N | Mean | Median | S.D. |
| *Industry-Level Sales Uncertainty* | | | | | | |
| Quarter *t*-3 |  |  | 668,786 | 0.514 | 0.461 | 0.308 |
| Quarter *t*-2 |  |  | 687,835 | 0.522 | 0.467 | 0.313 |
| Quarter *t*-1 |  |  | 699,811 | 0.523 | 0.456 | 0.314 |
| Quarter *t* |  |  | 708,924 | 0.518 | 0.452 | 0.313 |
| Quarter *t*+1 |  |  | 604,194 | 0.506 | 0.442 | 0.308 |
| Quarter *t*+2 |  |  | 594,154 | 0.501 | 0.438 | 0.306 |
| Quarter *t*+3 |  |  | 574,420 | 0.499 | 0.433 | 0.306 |
| *Firm-Level Forecast Dispersion* | | | | | | |
| Quarter *t*-3 |  |  | 408,668 | 0.033 | 0.046 | 0.431 |
| Quarter *t*-2 |  |  | 439,571 | 0.036 | 0.044 | 0.445 |
| Quarter *t*-1 |  |  | 444,988 | 0.034 | 0.046 | 0.435 |
| Quarter *t* |  |  | 450,066 | 0.050 | 0.047 | 0.403 |
| Quarter *t*+1 |  |  | 369,447 | 0.046 | 0.047 | 0.413 |
| Quarter *t*+2 |  |  | 375,888 | 0.043 | 0.043 | 0.388 |
| Quarter *t*+3 |  |  | 369,011 | 0.044 | 0.044 | 0.375 |
| This table presents univariate evidence on sales and earnings uncertainty around the election quarter. *Industry-Level Sales Uncertainty* is measured as the standard deviation of Δ*logSALES* within an industry, where Δ*logSALES* is calculated as the natural logarithm of the change in sales in the current quarter relative to the same quarter of the prior year. *Firm-Level Forecast Dispersion* is measured as the standard deviation of analysts’ quarterly earnings forecasts scaled by the mean forecast. Quarter *t* refers to the election quarter, Quarter *t*-1 (*t*+1) refers to one quarter before (after) the election quarter, and so on. | | | | | | |

| **Table E.3** | | | | | |
| --- | --- | --- | --- | --- | --- |
| **Additional analyses** | | | | | |
| **Panel A: State versus gubernatorial elections** | | | | | |
| Dependent variable = *WARN* | | (1) | | (2) | |
| *Pre-State Election* | |  | | -0.002*** | |
|  | |  | | [-6.09] | |
| *Pre-Gubernatorial Election* | |  | | 0.000 | |
|  | |  | | [0.48] | |
| *State Election* | | -0.003*** | | -0.003*** | |
|  | | [-9.13] | | [-9.99] | |
| *Gubernatorial Election* | | 0.001 | | 0.001 | |
|  | | [1.33] | | [1.36] | |
| *Post-State Election* | |  | | 0.002*** | |
|  | |  | | [4.84] | |
| *Post-Gubernatorial Election* | |  | | -0.000 | |
|  | |  | | [-0.24] | |
| *Control variables* | | Included | | Included | |
| *Firm-state fixed effects* | | Included | | Included | |
| *Year fixed effects* | | Included | | Included | |
| *N* | | 6,035,775 | | 6,035,775 | |
| Adjusted *R*^2^ | | 0.160 | | 0.160 | |
| **Panel B: Size of layoffs** | | | | | |
| Dependent variable = Log(1+*Layoffs*) |  |  | (1) | | (2) |
| *Pre-State Election* |  |  |  | | -0.009*** |
|  |  |  |  | | [-8.48] |
| *State Election* |  |  | -0.011*** | | -0.013*** |
|  |  |  | [-10.93] | | [-12.01] |
| *Post-State Election* |  |  |  | | 0.009*** |
|  |  |  |  | | [7.26] |
| *Control variables* |  |  | Included | | Included |
| *Firm-state fixed effects* |  |  | Included | | Included |
| *Year fixed effects* |  |  | Included | | Included |
| *N* |  |  | 6,035,775 | | 6,035,775 |
| Adjusted *R*^2^ |  |  | 0.158 | | 0.158 |
| This table presents weighted least squares regression results on the probability of firms issuing WARN notices around state elections, after controlling for gubernatorial elections (Panel A) and on how state elections influence firms’ decisions regarding the size of layoffs (Panel B). Regressions are weighted by firm exposure to different states mentioned in their 10-K filings. *Pre-Gubernatorial Election* is an indicator variable that equals one for the quarter immediately preceding the gubernatorial election, and zero otherwise. *Gubernatorial Election* is an indicator variable that equals one for the gubernatorial election quarter, and zero otherwise. *Post-Gubernatorial Election* is an indicator variable that equals one for the quarter immediately following the gubernatorial election, and zero otherwise. *Layoffs* is the number of employees laid off as disclosed in WARN notices, which is set to zero for observations with missing information in WARN notices or those without WARN notices. All other variables are defined in the appendix. *t*-statistics in parentheses are calculated using robust standard errors clustered by firm-state. ***, **, and * denote results significant at the 1%, 5%, and 10% levels (two-tailed). | | | | | |

| **Table E.4** | | | | | | | | |
| --- | --- | --- | --- | --- | --- | --- | --- | --- |
| **Comparison of financial performance between delayers and non-delayers** | | | | | | | | |
|  | Non-delayers | | | | Delayers | | | |
| Variable | N | Mean | Median | S.D. | N | Mean | Median | S.D. |
| *ROA* |  |  |  |  |  |  |  |  |
| Quarter *t* | 14,951 | -0.005 | 0.007 | 0.061 | 16,519 | 0.001*** | 0.007 | 0.039 |
| Quarter *t*+1 | 14,838 | 0.002 | 0.007 | 0.042 | 16,395 | 0.005*** | 0.008 | 0.030 |
| Quarter *t*+2 | 14,740 | 0.006 | 0.009 | 0.032 | 16,219 | 0.004*** | 0.009 | 0.032 |
| Quarter *t*+3 | 14,646 | 0.006 | 0.009 | 0.033 | 16,099 | 0.006 | 0.009 | 0.035 |
| Quarter *t*+4 | 14,492 | 0.007 | 0.009 | 0.035 | 15,915 | 0.003*** | 0.008 | 0.036 |
| Quarter *t*+5 | 13,780 | 0.004 | 0.007 | 0.027 | 15,827 | 0.006*** | 0.009 | 0.030 |
| Quarter *t*+6 | 13,662 | 0.007 | 0.010 | 0.023 | 15,653 | 0.005*** | 0.010 | 0.039 |
| Quarter *t*+7 | 13,588 | 0.007 | 0.010 | 0.027 | 15,405 | 0.004*** | 0.009 | 0.044 |
| *Profitability* |  |  |  |  |  |  |  |  |
| Quarter *t* | 14,358 | 0.079 | 0.120 | 0.877 | 15,941 | 0.124*** | 0.114 | 0.474 |
| Quarter *t*+1 | 14,257 | 0.108 | 0.114 | 0.648 | 15,847 | 0.123** | 0.125 | 0.555 |
| Quarter *t*+2 | 14,191 | 0.121 | 0.128 | 0.709 | 15,668 | 0.128 | 0.129 | 0.566 |
| Quarter *t*+3 | 14,069 | 0.159 | 0.130 | 0.200 | 15,549 | 0.138*** | 0.127 | 0.390 |
| Quarter *t*+4 | 13,975 | 0.153 | 0.127 | 0.186 | 15,409 | 0.142*** | 0.118 | 0.259 |
| Quarter *t*+5 | 13,331 | 0.144 | 0.123 | 0.326 | 15,319 | 0.146 | 0.129 | 0.290 |
| Quarter *t*+6 | 13,206 | 0.154 | 0.132 | 0.345 | 15,146 | 0.151 | 0.129 | 0.303 |
| Quarter *t*+7 | 13,133 | 0.165 | 0.133 | 0.177 | 14,834 | 0.144*** | 0.126 | 0.265 |
| This table compares the financial performance of delayers versus non-delayers from the election quarter onward. We define delayers (non-delayers) as firms issuing WARN notices one quarter after the election (during the election quarter). *ROA* is measured as net income divided by total assets. *Profitability* is measured as earnings before interest, taxes, depreciation, and amortization divided by sales. Quarter *t* refers to the election quarter, Quarter *t*+1 refers to one quarter after the election quarter, and so on. ***, **, and * denote significance of the difference in means between delayers and non-delayers at the 1%, 5%, and 10% levels (two-tailed). | | | | | | | | |

| **Table E.5** | | | | | | | |
| --- | --- | --- | --- | --- | --- | --- | --- |
| **Distribution of layoffs without WARN notices by quarter—relative to the election quarter** | | | | | | | |
| Quarter = | -3 | -2 | -1 | 0 | 1 | 2 | 3 |
| # of layoff observations (*N*) | 23,672 | 22,064 | 20,520 | 21,921 | 23,483 | 20,754 | 18,713 |
| *N* without WARN notices | 5,237 | 4,624 | 4,966 | 6,971 | 6,964 | 5,939 | 5,418 |
| % without WARN notices | 22.12 | 20.96 | 24.20 | 31.80 | 29.66 | 28.62 | 28.95 |
| This table presents the distribution of layoffs without WARN notices by quarter—relative to the election quarter. Quarter 0 refers to the election quarter, Quarter -1 (1) refers to one quarter before (after) the election quarter, and so on. | | | | | | | |
